# Consensus recommendations on fasting during Ramadan for patients with kidney disease: review of available evidence and a call for action (RaK Initiative)

**DOI:** 10.1186/s12882-024-03516-y

**Published:** 2024-03-06

**Authors:** Yousef Boobes, Bachar Afandi, Fatima AlKindi, Ahmad Tarakji, Saeed M. Al Ghamdi, Mona Alrukhaimi, Mohamed Hassanein, Ali AlSahow, Riyad Said, Jafar Alsaid, Abdulkareem O. Alsuwaida, Ali A. K. Al Obaidli, Latifa B. Alketbi, Khaled Boubes, Nizar Attallah, Issa S. Al Salmi, Yasser M. Abdelhamid, Nihal M. Bashir, Rania M. Y. Aburahma, Mohamed H. Hassan, Mohammad R. Al-Hakim

**Affiliations:** 1 Seha Kidney Care, Tawam Hospital, Al Ain, UAE; 2 Department of Medicine, College of Medicine and Health Science, UAE University, Al Ain, UAE; 3 Endocrine Division, Tawam Hospital, Al Ain, UAE; 4 Department of Medicine, Tawam Hospital, Al Ain, UAE; 5 St. George Medical Center & McMaster University-Waterloo Campus, Kitchener, ON Canada; 6 Prince Abdulmajeed Dialysis Center, Jeddah, Saudi Arabia; 7 Department of Medicine, Dubai Medical College, Dubai, UAE; 8 Endocrine Section, Dubai Hospital, Dubai Health, Dubai, UAE; 9 Mohammed Bin Rashid University of Medicine and Health Science, Dubai, UAE; 10 Division of Nephrology, Jahra Hospital, Jahra, Kuwait; 11 Department of Nephrology and Medicine, Jordan Hospital and Medical Center Ibn Sina University for Medical Sciences, Amman, Jordan; 12 Nephrology department, Ochsner Medical Center, New Orleans, LA USA; 13 Department of Medicine, King Saud University, Riyadh, Saudi Arabia; 14 Seha Kidney Care, Abu Dhabi, UAE; 15 Ambulatory Healthcare Services - Abu Dhabi Healthcare Services, Abu Dhabi, UAE; 16 Department of Medicine, Ohio State University, Columbus, OH USA; 17 Nephrology Associates of Kentuckiana, University of Kentucky, Louisville, USA; 18 Department of Renal Medicine, The Royal Hospital, Muscat, Oman; 19 Nephrology Division, Internal Medicine Department -Faculty of Medicine, Cairo University, Cairo, Egypt

**Keywords:** Chronic kidney disease, Ramadan fasting, Transplantation, Dialysis, Risk mitigation

## Abstract

Ramadan fasting (RF) involves abstaining from food and drink during daylight hours; it is obligatory for all healthy Muslims from the age of puberty. Although sick individuals are exempt from fasting, many will fast anyway. This article explores the impact of RF on individuals with kidney diseases through a comprehensive review of existing literature and consensus recommendations. This study was conducted by a multidisciplinary panel of experts.

The recommendations aim to provide a structured approach to assess and manage fasting during Ramadan for patients with kidney diseases, empowering both healthcare providers and patients to make informed decisions while considering their unique circumstances.

## Introduction

Fasting during the month of Ramadan constitutes a significant Islamic religious practice, entailing abstinence from food, drinks, and any intentional consumption, such as smoking and medication, from dawn to sunset. Certain individuals, such as prepubertal children, menstruating and pregnant women, breastfeeding mothers, sick people, elderly individuals, and travelers, are exempted from this religious obligation (Surat 2 "Al-Baqarah," Ayat 184–185). However, many within these categories may still choose to fast, seeking to partake in spiritual experiences with their families and peers.

Ramadan falls on the 9th month of the Muslim lunar calendar (Hijra), which comprises 354 days, in contrast to the solar calendar (Gregorian) with 365 days. Consequently, Ramadan occurs 11 days earlier each year and completes a cycle of approximately 33 years. This variation in timing results in a change in the fasting duration based on the year and geographic location. In northern cities, fasting periods can range from just a few hours (less than six hours) during winter Ramadan to over 18 h during summer Ramadan.

Ramadan fasting (RF) involves alternating periods of fasting and refeeding. Understanding its impact on kidney physiology and kidney-related conditions is of utmost importance, particularly when considering the management of chronic kidney disease (CKD) in Muslim patients determined to fast during Ramadan, irrespective of their geographic location.

Few studies have examined the impact of RF on patients with various kidney diseases and the role of diverse risk factors in determining the fasting risk for individual patients with kidney diseases (see Table 1).

**Table 1:** Available literature for assessing risks of Ramadan fasting in kidney disease

Potential Risk Factor	Studies in CKD Pts	Studies in Non CKD Pts
**• Severity of renal insufficiency (eGFR Level)**	Limited	
**• Level of Proteinuria**	Very Limited	
**• Kidney Transplant**		
A. **First year post transplant**	Very Limited	
B. **After first year post transplant**	Limited	
**• H/o Kidney Stones**	Limited	
**• Hemodialysis**	Limited	
**• Peritoneal dialysis**	Very Limited	
**• Presence of HTN:**		
A. **Controlled**	None	Limited
B. **Not Controlled**	None	None
**• Heart Diseases:**		
A. **CAD**	None	Limited
B. **Heart Failure**	None	Limited
C. **Arrhythmia**	None	Limited
**• Duration of Fasting**	None	None
**• Ambient Temperature**	None	None
**• Physical Labor**	None	None
**• Frailty & Age**	None	Limited
**• Liver disease &/or other GI diseases**	None	Limited
**• Pregnancy**	None	Limited
**• Electrolytes abnormalities**	None	Limited

Owing to the obligatory nature of RF, randomized interventional trials are not feasible, as individuals deemed fit to fast must comply, making them ineligible for randomization into non-fasting groups. Consequently, all published studies on RF and the kidney are non-interventional observational studies, predominantly prospective but also include several retrospective analyses. Many of these studies employed fasting individuals as their own controls, whereas others used non-fasting control groups comprising patients who chose not to fast. Moreover, the majority of the studies were conducted at single centers, often involving small patient populations, with absence of studies evaluating the long term effects of Ramadan fasting.

Given the multifaceted functions of the kidneys, the glomerular filtration rate (GFR) serves as an overall assessment of kidney function [1]. However, studies assessing the effects of RF on kidney function have utilized different methods to estimate the GFR. Some studies relied solely on serum creatinine levels as GFR surrogates, while others employed formulas such as Cockcroft-Gault, MDRD, or CKD-Epi. One study used renal DTPA scans.

These factors, among others, contribute to the limited nature of the evidence from existing studies, making it challenging to establish comprehensive fasting recommendations. A task force comprising expert physicians from different countries was assembled to conduct a review of existing literature and reach a consensus. To draw from the extensive expertise of Ramadan and Diabetes (DAR) in this domain, two prominent members of the DaR joined the Ramadan and Kidney Diseases (RaK) task force.

### Methodology

The initial concept was conceived by the first three authors who convened in person on three occasions to lay the foundation for the project. Following these initial meetings, an international panel of experts was identified, comprising nephrologists, endocrinologists, and family medicine physicians with expertise in CKD and fasting during the Ramadan.

International experts were invited to participate in a series of virtual meetings held over the course of several months. During these meetings, potential risk factors associated with fasting kidney diseases patients during Ramadan were thoroughly reviewed, and specific tasks were distributed among the group members. The literature relevant to the impact of fasting on kidney function has been extensively reviewed to ensure that the recommendations are evidence-based and comprehensive.

After the literature review, the group conducted several meetings to assess progress and collaboratively drafted the recommendations presented in this consensus statement. The process was characterized by rigorous debate, deliberation, and consensus building among the panel members. The resulting recommendations are the culmination of this multidisciplinary effort intended to provide guidance to healthcare professionals, patients, and researchers in the field of kidney diseases and fasting during Ramadan.

### Effect of severity of renal insufficiency

Several studies have investigated the impact of RF on patients with CKD. The majority did not identify significant differences in the kidney function parameters between fasters and non-fasters during Ramadan, or when patients were compared with themselves before and after RF [2–10]. Some studies have suggested that RF might lead to moderate improvement in kidney function [11, 12]. Chowdhury et al. conducted an observational study in the UK with 19 h fasting duration involving stable patients with coexisting CKD Stage 3 and type 2 diabetes. Sixty-eight patients observed the fast, while 71 did not. They found no statistically significant differences in outcome measures or adverse events between the two group [9].

El-Wakil et al. [13] assessed the impact of RF on renal function in a cohort of 15 patients with CKD with an average glomerular filtration rate of 33 ml/min/1.73 m^2^ in comparison to six healthy controls. While there were no significant differences in the change in GFR between the two groups, they found that CKD patients demonstrated a rise in biomarkers associated with increased tubular cell injury (urinary N-acetyl-B-D- glucosaminidase “NAG”). Another study by Ekinci et al. [4] failed to show any significant differences in urinary neutrophil gelatinase-associated lipocalin (NGAL) between fasting and non-fasting CKD patients.

A limited number of studies have reported worsening renal function in some patients with CKD during RF, primarily observed in those with moderate to severe CKD [14–17]. However, most of these studies lacked a non-fasting control group for comparison purposes. For example, Mbarki et al. reported acute kidney injury (AKI) occurrences in 7 out of 60 patients (11%) with CKD stage 2–4, but without follow-up after Ramadan and lacking a control group [14]. Ansari et al. found worsening kidney function in four patients (14.3%), all of whom were in stage IV and V, although two patients exhibited improvement after Ramadan, resulting in persistent worsening of renal function in only 7% [14].

Baloglu et al. documented AKI in 27 of 117 patients (23%) with CKD stages 2 and 3, but with no follow-up after Ramadan and no control group [16]. Another prospective observational study involving 65 CKD patients fasting in Ramadan with stage 3 or worse CKD demonstrated 22 patients (33.8%) with AKI, as defined by an increase in creatinine of 26.5 umol/L or greater. Among these, 15 patients experienced AKI during Ramadan, 7 patients within three months after Ramadan, 8 patients exhibited subsequent improvement, and 14 continued with persistent reduction in kidney function [17].

A systematic review conducted by Bragazzi [18] identified 26 pertinent studies, of which 5 were specifically focused on CKD non-dialysis and dialysis patients. These studies collectively revealed that RF is generally well tolerated among patients with CKD, albeit with certain caveats. Furthermore, Bragazzi [19] conducted another meta-analysis encompassing six studies involving 350 patients with CKD during Ramadan and monitored changes in eGFR. Of these studies, only two were performed in patients with CKD, and the rest were in transplant recipients. These two meta-analyses were conducted in 2014 and 2015, respectively. More recently, Bello et al. [20] published their protocol for a systemic review in 2019, but the final results have not yet been reported.

In Summery, Available data suggests a progressive increase in the risk of AKI with CKD severity [13, 17]. Stages 1 and 2 show a low risk, stage 3 exhibits a moderate risk, while stages 4 and 5 pose a high risk.

### Effect of presence of proteinuria in CKD patients

While some studies have identified the presence of proteinuria as an independent risk factor for renal dysfunction in CKD patients with RF [2], this association has not been consistently confirmed by other studies. Given that these studies were not originally designed for this purpose, we propose considering only nephrotic-range proteinuria as an additional risk factor for CKD in patients with RF.

### Effect of Ramadan fasting on pregnant women with CKD

No available study addressed this question. However, observational studies and meta-analyses have assessed the effect of RF on pregnancy without CKD and revealed no harm to pregnant women or their fetuses by RF [21–25].

On the other hand, it is well known that pregnant women with CKD have an increased risk of adverse maternal and infant outcomes in general [26–30], and that pregnancy could cause a flare-up of certain kidney diseases [31].

Taking these data as well as the fact that it is permissible for healthy pregnant and breastfeeding women to break the fast in Ramadan due to concerns about their own health or that of their children [32–35], we recommend that pregnant women with CKD should not fast during Ramadan.

### Frailty and cognitive function and Ramadan fasting

Frailty, a syndrome predominantly associated with aging, increases susceptibility to adverse health outcomes [36]. Although we could not find studies specifically assessing the impact of RF in frail CKD patients, the findings from related research are instructive.

Kara et al. observed that advanced age was an independent predictor of renal function deterioration in their study [5]. Bakhit et al. reported through multivariate analysis that more advanced CKD stage, higher baseline systolic blood pressure, and younger age were all independently associated with worsening kidney function [17], which might contradict the results of the study by Kara et al.

Similar to the provision for pregnant women, we advise that frail individuals encounter difficulties in RF to abstain from fasting even in the absence of any underlying medical condition.

### Ramadan fasting for kidney transplant recipients

Several studies have investigated the impact of RF on kidney transplant recipients [37–49], and many systematic or mini-reviews have been conducted on the same topic [18, 50–53].

The studies were observational in nature with small sample sizes. Some compared kidney transplant recipients with themselves (pre-Ramadan vs during/post-Ramadan) others had a control group from kidney recipients not fasting during Ramadan. The majority of kidney recipients in these studies had good and stable baseline renal function and fell into CKD stages 1-3T. The risk of acute rejection is generally higher during the first few months post transplantation, and most of these studies excluded kidney recipients who had not completed their first year post-transplantation [40, 42–46]. Only one small study has examined the safety of fasting during the initial year of transplantation. The study included 14 patients who insisted on fasting during Ramadan in 1997, spanning from one to seven months post-transplantation [49]. All patients were on azathioprine and prednisolone and maintained good kidney function (mean plasma creatinine level of 95 ± 15 μmol/L).

These studies (including the last one [49]) as well as the systemic reviews did not reveal any significant changes in kidney function and concluded that RF is safe for patients with stable kidney function.

Ghalib et al. [41] studied whether repeated RF could adversely affect kidney function in kidney transplant recipients. They calculated the eGFR (using the Cockcroft-Gault formula) in 35 transplant patients in the fasting group and 33 in the non-fasting control group both before and after three consecutive Ramadans (2004, 2005, and 2006). They found that RF did not adversely affect kidney function, and no significant change was observed in the eGFR after the third Ramadan compared with the baseline or with the control group. Of interest, five patients in the fasting group had transplants for less than 1 year. Another study yielded similar results and examined kidney transplant recipients with diverse post-transplant periods, including some individuals who had undergone transplantation less than one year prior. However, the study did not provide a precise count of recipients in this particular subgroup. Nevertheless, they did provide the mean post-transplant period, which averaged 2 years, with a range extending from 0.6 to 6.3 years [37].

Based on this limited evidence, we recommend against fasting in the first year after kidney transplant surgery. However, after one year, if a patient exhibits good kidney function (CKD stage I to stage III), fasting does not disrupt their immunosuppressant medication schedule, and has maintained stable kidney function over the previous 3 months, they have the option to decide whether to fast during Ramadan. If a patient chooses to fast, it is crucial to do so with close medical supervision. We recommend monitoring kidney function within the four weeks preceding Ramadan to ensure stability. We also advise repeating the laboratory tests one week after starting fasting to confirm safety. Patients should maintain adequate fluid intake throughout the night, and if they inadvertently forget to take their immunosuppressive medications during the sahoor (pre-dawn meal), it is imperative for them to break their fast on that particular day to take these medications.

### Ramadan fasting for patients on dialysis treatment

Ramadan fasting poses notable challenges for patients undergoing dialysis treatment such as hemodialysis or peritoneal dialysis.

In predialysis CKD patients, the primary medical objective is to preserve the remaining kidney function, and other health considerations also play a significant role. However, for dialysis patients already undergoing kidney replacement therapy, where native kidney function is essentially nonexistent or minimal, the focus shifts towards managing other associated health concerns. Notably, the available studies examining the effects of RF on dialysis patients are limited in number and scope.

### Maintenance hemodialysis treatment

Studies addressing RF in hemodialysis patients, in general, did not show significant risks [54–61]. However, one Saudi study [59] showed that the fasting group had higher mean interdialytic weight gain (IDWG) by 0.62 kg compared to the non-fasting group. Other studies observed no significant change in dry weight during Ramadan and no increased incidence of pulmonary edema, hypertension, or intradialytic hypotension [55, 56], while a study from Malaysia observed a decrease in IDWG [60].

Another potential risk factor in this group was hyperkalemia. Patients with end-stage kidney disease (ESKD) are more likely than normal controls to develop hyperkalemia after 16 h of fasting. This phenomenon has been attributed to insulinopenia and a diminished response to epinephrine [62]. One study [59] showed that the fasting group exhibited elevated potassium levels when compared to the non-fasting group; however, there was no discernible difference in potassium levels within the fasting group before and during Ramadan. Other studies have found no significant variations in tests in hemodialysis patients during RF [54, 56].

A multicenter, prospective observational study conducted by Adanan et al. [60] revealed a significant improvement in handgrip strength during Ramadan, which continued after Ramadan in patients on maintenance hemodialysis who observed fasting.

### Peritoneal dialysis (PD)

We identified a single study conducted by Al Wakeel et al. in Riyadh, Saudi Arabia, during the fasting period of Ramadan in 2009, spanning 14 h in duration [63]. They modified the PD schedule to allow for no fluid exchange during fasting hours. A total 31 PD patients wished to fast. They reported no serious morbidity or mortality and concluded that the most stable patients on PD can fast.

It is important to note that all the studies mentioned above lacked randomization, which is common in research related to RF. This could introduce a significant risk of selection bias since it is probable that healthier and more physically fit patients are more inclined to choose fasting. It is crucial to exercise caution when generalizing the results of these studies to all patients on dialysis. Patients undergoing dialysis typically have a multitude of comorbidities that are likely not adequately represented in these studies. Therefore, we recommend classifying patients on dialysis as high-risk individuals for fasting. This implies that dialysis patients are generally discouraged to fast; if they insist on fast, they should do so under medical supervision. With prior positive fasting experiences, a patient's risk level may shift from high to moderate. This approach prioritizes patient safety given the complexities and risks associated with dialysis and the presence of multiple comorbid conditions in these individuals.

### Hypertension in CKD patients who fast during Ramadan

The available literature on this specific topic is limited. We could not find controlled studies specifically aimed at assessing the effects of fasting during Ramadan on CKD patients in the presence or absence of hypertension versus no hypertension. A small prospective self-controlled study from Egypt examining RF encompassed both hypertensive patients with and without CKD. While the mean serum creatinine level increased from 1.06 mg/dL before RF to 1.11 mg/dL post-Ramadan, the level of eGFR did not change significantly. Systolic and diastolic blood pressures (systemic and central) decreased significantly after RF, regardless of CKD status, suggesting that both CKD and non-CKD hypertensive patients can safely observe RF [64].

Other studies have reported in their analysis a significant relationship between the presence of hypertension in CKD patients and the worsening of kidney function during RF [16, 17]. Another study failed to confirm this result [5].

On the other hand, several studies in non-renal hypertensive subjects assessed the influence of RF on blood pressure (BP) control and found no statistical difference between fasting and non-fasting periods in 24 h BP monitoring [65–68]. A large cross-sectional study conducted among 1118 hypertensive patients showed that RF resulted in small but significant improvements in most biochemical parameters [69]. Other studies have reported improved BP control due to RF [70–72]. Two systematic reviews concluded the same [73, 74]. Another meta-analysis suggested beneficial effects of RF on BP independent of changes in weight, total body water, and fat mass, supporting recommendations for some governmental guidelines that describe RF as a safe religious practice with respect to BP [75].

Patients with uncontrolled hypertension were excluded from most studies. However, as they require multiple doses during the daytime, it is recommended that they be counseled against fasting [76].

### Ramadan fasting for CKD patients with cardiovascular diseases

There is limited evidence available on the effects of Ramadan fasting in CKD patients with cardiac disease versus those without cardiac disease.

NasrAllah et al. observed adverse cardiovascular events in six patients (11.5%) in the fasting cohort during the month of Ramadan compared with only one patient (1.9%) in the control group (*p* = 0.036) and were associated with an increase in serum creatinine after one week of fasting and the presence of pre-existing cardiovascular disease [10].

Studies on cardiac patients without CKD have generally found fasting feasible [77–80]. In an observational study, the NYHA functional classification remained unchanged in 92% of the patients who underwent RF [77]. Another study found no significant increase in heart failure hospitalizations during the Ramadan [79]. Two observational studies on patients with chronic coronary syndrome (CAD) [81, 82] found that RF was not associated with increased cardiac mortality or morbidity. The impact of RF on cardiovascular risk factors in patients with stable coronary heart disease is generally favorable [83, 84]. Temizhan et al. [85] found no significant differences in the incidence of acute coronary syndrome (ACS) between the months before, during, and after Ramadan. One study demonstrated that patients who underwent RF within three months of percutaneous coronary intervention had a higher incidence of significant cardiac events than those who did not [86]. Patients with advanced heart failure requiring frequent hospitalization, unstable cardiovascular disease, or arrhythmia need further evaluation prior to fasting, given the risk of complications [76].

Available data regarding CKD patients with cardiac disease are limited, which makes it challenging to establish recommendations. We strongly advocate a thorough evaluation by both nephrologists and cardiologists to collectively determine the safety of RF for these patients.

### Ramadan fasting for CKD patients with liver diseases

The influence of RF on individuals with both CKD and concurrent liver disease is complex and understudied. There is an almost complete absence of studies addressing patients with CKD and liver disease during RF.

However, a review of the impact of RF on individuals with liver diseases [87] found that patients with stable liver function and chronic hepatitis B and C can safely fast during Ramadan. For patients with liver cirrhosis, an extensive evaluation is crucial. Patients with Child–Pugh class A liver cirrhosis can participate in fasting, while Child–Pugh class B cirrhosis patients require evaluation based on age and comorbidities. Fasting is generally discouraged in patients with Child–Pugh C liver cirrhosis because of the high risk of complications [88–91]. For non-alcoholic fatty liver disease, RF has shown favorable outcomes in non-alcoholic fatty liver disease [92, 93]. No studies have investigated the effect of fasting on patients with liver cancer [88]. Limited studies have examined the effects of RF on liver transplant recipients [94, 95].

Given the complexity and diversity of liver diseases, it is essential for patients with CKD and liver diseases to undergo a comprehensive evaluation by both nephrologists and gastroenterologists.

### The impact of Ramadan fasting during hot summers on CKD patients

Higher ambient temperatures are generally associated with an increased incidence of AKI, which can occur due to hypovolemia or severe heat exposure-induced conditions, such as rhabdomyolysis and inflammation [96–98]. CKD patients observing RF and exposed to a high temperature could be at an increased risk of heat stress, AKI, and stone formation.

The existing literature lacks direct investigation to address this question. Nonetheless, a reasonable conclusion is that CKD patients in hot climates during RF may have an increased risk of kidney complications, depending on the level of temperature and duration of exposure.

### The impact of Ramadan fasting on CKD patients with unstable kidney function or acute illness

The existing literature lacks direct investigations addressing this question.

Most studies have excluded CKD patients with acute kidney injury or individuals experiencing acute illnesses, based on the assumption that RF is unsafe for them. We recommend that patients with CKD with unstable kidney function or those currently dealing with acute illnesses refrain from fasting.

### The impact of Ramadan fasting on CKD patients with nephrolithiasis

Data from epidemiological and clinical studies have demonstrated that increased urine volume achieved by high fluid intake exerts an efficacious preventive effect on the onset and recurrence of urinary stones [99].

One might presume that RF with no fluid intake during the daytime would predispose susceptible patients to an increased risk of stone formation and renal colic attacks.

The available literature reveals a trend toward RF not increasing the risk of developing urinary stones. However, this remains controversial. Most studies have failed to find a relationship between RF and urinary stones [100–107]. A few studies reported an increased number of renal colic admissions during or after Ramadan [108, 109] There was an association between RF and the incidence of renal colic admissions.

Two recent systematic reviews of the impact of RF on renal stone formation are available. The first (2020) included five studies [110], and the second (2021) identified 10 observational studies [111]. Both studies concluded that RF is unlikely to significantly increase the risk of renal stones.

Owing to limitations in the literature, it is not possible to form a strong opinion on whether fasting during Ramadan affects stone formation. It is also unclear whether RF in summer and hot climate areas adds an additional risk of stone formation.

### The impact of Ramadan fasting on patients with diabetes and CKD

In diabetic patients with CKD, managing RF adds complexity. To ensure informed decision-making and enhance patient safety, we strongly advise healthcare providers to utilize the established Diabetes and Ramadan (DAR) risk calculator [112]. This tool considers multiple clinical parameters, including renal function, to evaluate fasting risks and offer personalized recommendations.

### Group recommendations “shown in Fig. 1”

**Fig. 1:**
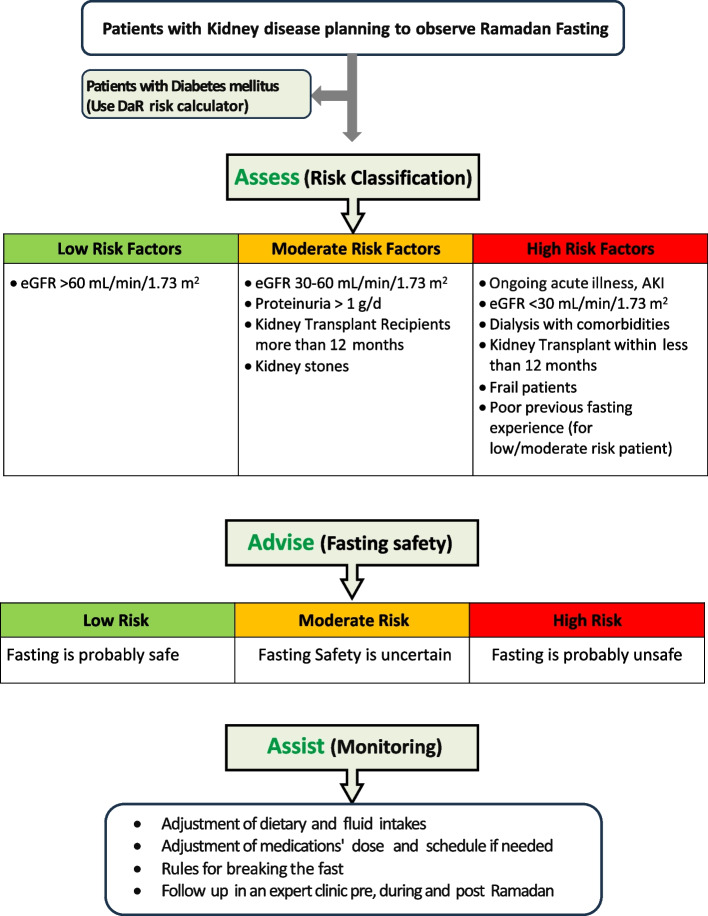
RaK clinical assessment tool (3A’s: Assess, Advise & Assist): This figure delineates the RaK recommendations, organized into three steps. The initial step involves the pre-Ramadan assessment to classify the risk of fasting (risk classification), determining whether fasting is advised or not (second step). If a patient, identified with moderate or high risk, chooses to fast, the subsequent step (third) involves assisting them to do it safely, under close monitoring

In light of the review of existing evidence, along with the collective expertise of participating professionals, this task force has developed the first Ramadan and Kidney Disease (RaK) consensus recommendations. These recommendations are designed to provide a structured and as much as possible, evidence-based approach to pre-Ramadan assessment and risk classification, during Ramadan evaluation, and post-Ramadan follow-up for individuals with CKD (Tables 2 and 3). Many social factors could influence these recommendations (availability of air conditioning, patient's profession, housing…) should be taken in consideration. By addressing the challenges and considerations associated with CKD and RF, these recommendations aim to empower both healthcare providers and patients to make informed decisions regarding fasting during the sacred month.

**Table 2:** Recommendations for CKD patients planning to fast

**Pre- Ramadan Assessment:**	
**Schedule clinic visit:** at 1 to 3 months before Ramadan	- Review previous fasting experience
	- Evaluate for frailty [113]
	- Evaluate clinical and other comorbid conditions
	- Assess wellness for fasting and evaluate potential risk factors
	- Measure of BP, weight / BMI
	- Check basic laboratory investigations: creatinine and electrolytes, eGFR, CBC, blood glucose, calcium, phosphorus levels, protein-to-creatinine ratio, drug levels in patients on tacrolimus or cyclosporine (whenever indicated)
	- Adjust medications to suit fasting hours, detailing the dosing schedule for Ramadan - Substitute short-acting medications with long-acting formulations (e.g., tacrolimus) or patch-based options (eg clonidine), if available - Gradually taper off medications prone to rebound effects (e.g., oral clonidine) if patches aren't an available - Inform the patient about the need to adjust the timing for measuring calcineurin inhibitor (tacrolimus or cyclosporine) trough levels to align with their revised schedule
	- Dietary adjustment and water / fluid intake during Ramadan: - To eat balanced diet that fits patient’s health - To avoid potassium rich food (dried fruits, banana, fruit juice,..) if hyperkalemia is a concern - To break the fast gradually: Begin with a cup of water, followed by light meal, then to have the main meal post Taraweeh prayer - Should not skip Sahoor “pre-dawn meal” - To stay hydrated: to drink at least 1.5 to 2.5 L of water throughout the night, avoiding excessive intake at once. Space out your consumption
	- Discuss physical (type and timing) activity during Ramadan
	- Refer to other subspecialty clinics for the evaluation of relevant fasting risk factors (nephrology, cardiology, endocrinology, and others), whenever indicated
	- Classify patient risk and recommend accordingly. (see Fig. 1)
	- Monitoring during Ramadan fasting is advised for all patients
	- Discuss the rules for early termination of the fast (Table 3)
**During- Ramadan Assessment:**	
**Schedule clinic visit:** 1st week of Ramadan for moderate and high-risk patients who opted to fast	- Ask about tolerating fasting and any issues encountered
	- Check BP, weight / BMI, blood glucose
	- Basic laboratory investigations: Creatinine and electrolytes, eGFR, CBC, and drug trough levels in patients on tacrolimus or cyclosporine (be careful about the time of the test)
	- Review medications and if patient is following the recommended adjustments
	- Advise about dietary adjustment, water / fluid intake
	- Review type and timing of physical activity during Ramadan
	- Review the rules for early termination of the fast
**Post- Ramadan Assessment:**	
**Schedule clinic visit:** at 1 then 3 months after Ramadan	- Evaluate the fasting experience and any issues encountered
	- Measurement of BP, weight / BMI, blood glucose
	- Basic laboratory investigations: HbA1c, creatinine, and electrolytes, eGFR, CBC, lipid panel, drug levels in patients on tacrolimus or cyclosporine
	- Medications dose adjustment and schedule (resume pre-Ramadan)
	- Evaluate the comorbid conditions post Ramadan

**Table 3:** Rules for early termination of the fast

◦ Any acute illness causing (fever, diarrhea, exhaustion, vomiting …)
◦ Any condition requiring hospitalization (trauma, suspected cardiovascular event)
◦ AKI, noted on laboratory investigations
◦ Hyperglycemia Blood glucose > 300 mg/dL (16.6 mmol/L), or hypoglycemia Blood glucose < 70 mg/dL (3.9 mmol/L), even in asymptomatic patients
◦ Kidney transplant patient missed taking his immunosuppression medications at Sohour

In summary, the RaK Initiative, a collaboration of nephrologists, endocrinologists, and family physicians, sheds light on the intricate relationship between RF and kidney disease. Our recommendations emphasize the importance of pre-Ramadan evaluation, personalized guidance, and risk assessment. We considered factors such as eGFR, proteinuria, transplant status, and comorbidities to help healthcare providers decide on fasting eligibility.

While evidence of RF in the context of kidney disease is limited, our work underscores the need for tailored care and monitoring to ensure that individuals with kidney disease can safely participate in RF and harmonize their spiritual observance with optimal healthcare practices. Further research is needed to expand our understanding and refine our recommendations to prioritize patient safety and well-being.

### Recommendations for future research

The road ahead necessitates critical, multidisciplinary collaborations to design and execute high-quality clinical studies delving into the diverse effects of fasting on patients with renal diseases. Standardizing acceptable endpoints and assessment methodologies, coupled with comprehensive details regarding fasting duration, environmental influences, and social factors, could significantly enhance the interpretation of study outcomes.

Presently, there is a notable dearth or extremely limited studies examining the impact of Ramadan fasting on kidney disease patients with various accompanying risk factors (refer to Table 1). For instance, investigations are needed to explore the effects of RF on CKD patients with heart failure, those with ischemic heart disease, individuals with hypertension, among other specific conditions. Expanding research into these specific subgroups within the CKD patient population during RF could provide invaluable insights.

## Data Availability

No datasets were generated or analysed during the current study.
